# Correction: Al Khatib et al. Inhaled Medicines for Targeting Non-Small Cell Lung Cancer. *Pharmaceutics* 2024, *15*, 2777

**DOI:** 10.3390/pharmaceutics16091136

**Published:** 2024-08-28

**Authors:** Arwa Omar Al Khatib, Mohamed El-Tanani, Hisham Al-Obaidi

**Affiliations:** 1 School of Pharmacy, University of Reading, Reading RG6 6AD, UK; 2 Faculty of Pharmacy, Al Ahliyya Amman University, Amman 19111, Jordan; 3 College of Pharmacy, RAK Medical and Health Sciences University, Ras Al Khaimah P.O. Box 11172, United Arab Emirates

In the original publication [1], Figure 1 was created by an artificial intelligence (AI) tool but without disclosing the information in the Material and Methods section. The corrected Figure 1 appears below. The authors state that the scientific conclusions are unaffected. This correction was approved by the Academic Editor. The original publication has also been updated.

## Figures and Tables

**Figure 1:**
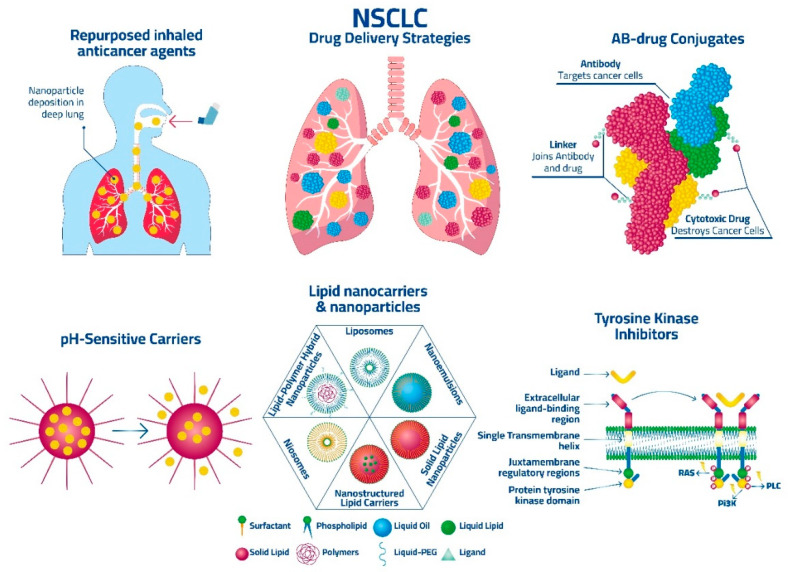
Examples of current drug delivery strategies for the treatment of NSCLC.
